# Circulating Amino Acid Changes Three Years After Bariatric Surgery

**DOI:** 10.3390/metabo15050297

**Published:** 2025-04-30

**Authors:** Ina Maltais-Payette, Fannie Lajeunesse-Trempe, Mélanie Nadeau, Léonie Bouvet-Bouchard, Frédéric Simon Hould, Laurent Biertho, André Tchernof

**Affiliations:** 1 Heart and Lung Institute, Laval University, Quebec, QC G1V 4G5, Canada; 2 School of Nutrition, Faculty of Agricultural and Food Sciences, Laval University, Quebec, QC G1V 0A6, Canada; 3 Faculty of Pharmacy, Laval University, Quebec, QC G1V 0A6, Canada; 4 Department of Surgery, Faculty of Medicine, Laval University, Quebec G1V 0A6, QC, Canada

**Keywords:** amino acids, bariatric surgery, obesity

## Abstract

Background and objective: Studies using metabolomics to study bariatric surgery have shown that amino acids are one of the most changed groups of metabolites after the intervention. However, the surgery-related variation in individual amino acids, as well as the long-term impact and the differences between the types of surgeries, have been poorly studied. The aim of this study was to investigate the changes in circulating amino acids after three types of bariatric surgery up to 36 months after the intervention. Methods: We studied 63 participants diagnosed with T2D at baseline, who received either a sleeve gastrectomy, a Roux-en-Y gastric bypass or a biliopancreatic diversion with duodenal switch. We measured the concentrations of 16 circulating amino acids in fasting plasma before the surgery as well as after 4, 12, 24 and 36 months via liquid chromatography coupled with mass spectrometry (LC-MS/MS). Results: Eleven circulating amino acids were significantly modified by bariatric surgery. Glutamate, leucine and isoleucine showed the greatest decrease. Most of the changes in circulating amino acids occurred within 1 year of the operations. Only one measured plasmatic amino acid (threonine) had a significantly different change pattern according to surgery types. In repeated-measure correlations, changes in circulating amino acids were significantly associated with changes in adiposity and metabolic markers. Conclusions: Bariatric surgery changes the levels of most circulating amino acids, and the effect occurs in the short term without major differences between surgery types. The mechanisms explaining these changes are not elucidated but likely include modifications in amino acid metabolism.

## 1. Introduction

Since 2009, attention has been given to the association between obesity and circulating amino acids [1]. A meta-analysis of this literature shows that obesity is associated with higher circulating levels of leucine, isoleucine, valine, alanine, lysine, glutamate, proline, ornithine, tyrosine and phenylalanine, as well as with lower levels of citrulline, serine, glycine and glutamine [2]. We and others have demonstrated that circulating levels of glutamate were more strongly associated with abdominal and visceral adiposity than other amino acids [3,4,5,6]. The pathophysiology underlying the associations between circulating amino acids and adiposity is not yet fully understood. Moreover, little is known about the changes in circulating amino acids after weight loss.

Bariatric surgery is the surgical modification or rearrangement of the digestive tract to induce weight loss. It is currently recognized as the most effective treatment to induce sustainable weight loss in patients living with severe obesity [7]. However, weight regain after bariatric surgery occurs for some patients, with a wide range of reported prevalence, from 3.4% to 38%, depending on the definition used for weight regain, the type of surgery and the follow-up time [8]. This intervention is recommended for individuals with a body mass index (BMI) greater than 35 kg/m^2^ and those with a BMI between 30 and 34.9 kg/m^2^ living with type 2 diabetes, or in whom non-surgical treatment has failed to induce weight loss and an improvement in metabolic co-morbidities [9]. Some bariatric surgery procedures are traditionally viewed as restrictive, such as sleeve gastrectomy (SG), and others as both restrictive and malabsorptive, such as Roux-en-Y gastric bypass (RYGB) and biliopancreatic diversion with duodenal switch (BPD-DS). Bariatric surgeries have been shown to improve metabolic health beyond what is expected from the weight loss that they induce, through molecular mechanisms that are not fully elucidated [10].

Studies investigating changes in metabolomic profiles following bariatric surgery have shown that circulating amino acid concentrations are modified by these interventions [11]. However, changes beyond 1 year after surgery and the differences between surgery types have rarely been documented. Moreover, most studies have examined only a limited number of amino acids.

The aim of this study was to characterize the changes in fasting concentrations of circulating amino acids up to 3 years after bariatric surgery and the differences between surgical modalities.

## 2. Materials and Methods

### 2.1. Sample

Participants were recruited at the Quebec Hearth and Lung Institute in Canada. The sample was selected from a larger study whose inclusion criteria were severe obesity, a type 2 diabetes diagnosis, eligibility for bariatric surgery and the ability to consent. The exclusion criteria were contraindications to bariatric surgery, i.e., a BMI under 35 kg/m^2^; an age under 18 or over 65 years; digestive diseases; pregnancy; liver cirrhosis; albumin deficiency or anterior bariatric surgery.

We selected 63 patients to create 3 groups of 21 participants who received either SG, RYGB or BPD-DS, as described elsewhere [12]. The groups were matched for age and sex, but could not be matched for pre-operative BMI due to patients receiving RYGB being smaller on average. Patients were followed up before surgery as well as 4, 12, 24 and 36 months after the operation. At each follow up, anthropometric measurements were taken, blood pressure was measured and blood was collected. The homeostasis model assessment for insulin resistance (HOMA-IR) index was calculated from fasting plasma glucose and insulin concentrations [13]. At the time of surgery, a liver biopsy sample was collected and analyzed by a trained pathologist to determine liver histological features.

### 2.2. Amino Acid Measurements

Fasting blood samples were collected in EDTA tubes and plasma was preserved at −80 °C until analysis. For extraction, 100 μL of plasma was mixed with 400 μL of a chloroform/methanol (2:1) mixture. A total of 100 μL of methanol phase and 200 μL of chloroform phase were collected in separate tubes, dried using SpeedVac (Savant SPD140DDA, Thermo Fisher Scientific, Waltham, MA, USA) and kept at −80 °C until injection. On the day of injection, the dried methanol phase was reconstituted in 100 μL of a methanol/water solution acidified with 0.1% formic acid, followed by the addition of 100 μL acetonitrile. The reconstitution solvent was supplemented with deuterium-labelled glutamate, leucine, isoleucine and tyrosine, because these amino acids were of particular interest based on our previous work [14,15,16]. The reconstituted samples were filtered (InnoSep Spin Nylon 0.2 µm, Canadian Life Science, Peterborough, ON, Canada) and placed into LC vials. Pooled samples, blanks and amino acid standards (Millipore Sigma, Saint-Louis, MO, USA) were used for quality control and identification. The prepared samples were analyzed on a UHPLC Vanquish coupled to a Fusion Tribrid (both from Thermo Fisher Scientific, Waltham, MA, USA). The samples were kept at 4 °C in an autosampler before being injected (2 µL) on a Acquity BEH Amide (1.7 µm, 2.1 × 150 mm) column (Waters, Mississauga, ON, Canada) kept at 40 °C using a gradient (Supplementary Table S1) of A: 50% acetonitrile and B: 95% acetonitrile, both with 10 mM ammonium formate, at pH 3. The data were acquired in both positive and negative modes using the DDA method, acquiring data between 50 and 1000 Da at a mass resolution of 60,000 for MS1 and 15,000 for MS2. Sixteen amino acids were measured as part of a broader untargeted metabolomics profile. Their identification was performed using their mass, retention time, fragmentation pattern and a comparison with standards (when possible). Details on the identification of the 16 amino acids measured are presented in Supplementary Table S2.

### 2.3. Dietary Amino Acids

The dietary intakes of a subgroup of patients were measured before the surgery (*n* = 21) as well as at 4 months (*n* = 22) and 12 months (*n* = 23) after surgery through a self-administered, web-based food frequency questionnaire [17]. It included 136 food items or food clusters for which patients were asked to specify their intake in terms of frequency and portion size over the last month. This web-based questionnaire was previously validated in a sample of French-speaking Canadians [17]. Only complete questionnaires were considered.

### 2.4. Statistical Analysis

The baseline characteristics of the sample were compared between surgery types using a Kruskal–Wallis test. We examined the changes in anthropometric and metabolic variables, as well as circulating amino acids, after bariatric surgery using mixed linear models. The variables were transformed when needed to comply with the test assumptions. First, to examine how the variables changed after surgery, we used a mixed linear model with time as a fixed parameter. The differences between specific time points were evaluated by a Tukey HSD post hoc test. Second, to examine the effect of surgery type, we ran a mixed linear model with time, surgery type and time-by-surgery interaction parameters. Finally, to determine the changes independent of weight loss, we ran a third mixed linear model with time, surgery and BMI as fixed effects.

To determine the association between the change in circulating amino acids and the change in anthropometric variables, we used repeated-measure correlations [18]. This method measures intra-individual association between variables of interest.

We identified individuals who regained weight as those with more than 10 kg of weight regain after reaching nadir (lowest weight) [8]. We compared the baseline characteristics as well as the circulating amino acids between participants who regained weight and those who maintained their weight loss using a *t*-test.

We computed the correlation between the circulating levels and dietary intakes of amino acids using Spearman’s rank correlations. Based on the work by Welch et al. [19], we excluded 1 observation that appeared aberrant based on the ratio of energy intake to basal metabolism rate (calculated using the Mifflin St-Jeor equation).

A *p*-value below 0.05 was considered significant. For analyses of the 16 amino acids, we adjusted the *p*-values using Bonferroni’s method. We did not adjust the *p*-value for multiple testing in the weight regain analysis, as it was exploratory, and statistical power was already limited.

## 3. Results

### 3.1. Sample Characteristics

The pre-operative characteristics of the sample are presented in Table 1 by surgery type. Overall, the median (Q1–Q3) age was 51.5 (44.0–55.3) years, the median body mass index (BMI) was 44.2 (40.8–48.6) kg/m^2^ and the majority (67%) of participants were female. As per the study design, there was no significant differences between the surgical groups for age and sex (Table 1). All patients had type 2 diabetes, with a median diabetes duration of 5 (1.5–11) years. In addition, 46 participants (73%) had hypertension and 36 participants (57%) had dyslipidemia.

The only pre-operative variables that were significantly different between surgery groups were BMI and waist circumference (WC) (Table 1). In the multiple comparison analysis, BMI was significantly lower in the RYGB group compared to the SG (adjusted *p*-value = 0.0235) and BPD-DS groups (adjusted *p*-value = 6.8 × 10^−06^). Waist circumference was significantly lower in the RYGB group compared to the BPD-DS group (adjusted *p*-value = 0.0171).

Among the 16 circulating amino acids measured, only tryptophan was significantly different between the surgical groups at baseline. Its circulating level was significantly higher in the SG group versus the BPD-DS group (adjusted *p*-value = 0.0364).

As expected, BMI decreased significantly after surgery (*p* < 0.0001). The multiple comparison analysis showed that BMI decreased significantly up to 12 months post-surgery and remained stable afterward. There was a significant interaction of BMI as a function of time and surgery; BMI decreased more acutely in the DBP-DS group compared to the SG and the RYGB groups. Most cardiometabolic parameters were also improved after surgery. For example, the HOMA-IR index, fasting triglycerides and systolic blood pressure all decreased significantly up to 4 months after surgery (all adjusted *p*-values < 0.001) and remained stable afterward.

### 3.2. Changes in Circulating Amino Acids

**Figure 1:**
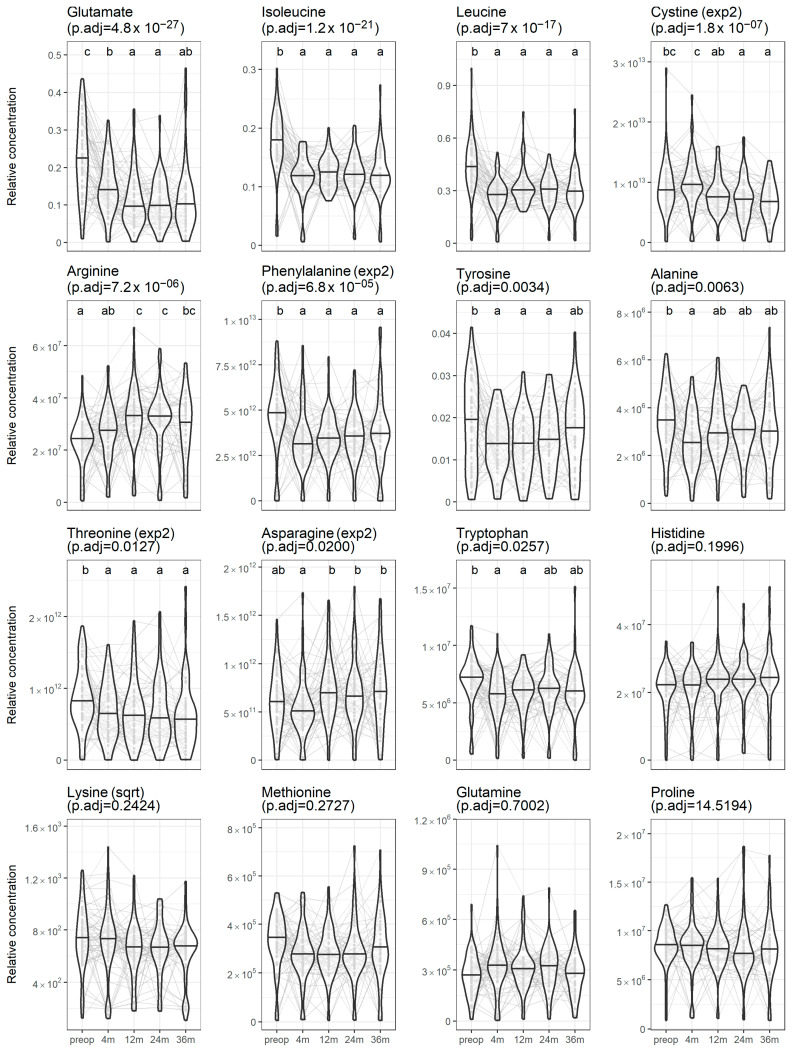
Changes in circulating amino acids up to 36 months after bariatric surgery. Circulating amino acids were compared across time points using a mixed model with time as a fixed parameter and individuals as a random parameter. Letters express the results of the Tukey HSD post hoc test; different letters mean that the time points were significantly different. The variables were transformed when needed to comply with the test assumptions. The concentration of amino acids is expressed in relative values. The violin plots show the distribution of circulating amino acids at each time point; the middle lines correspond to the median and the grey points and lines are individual observations. The *p*-values were adjusted using Bonferroni’s correction.

Out of the 16 circulating amino acids measured, 11 changed significantly after bariatric surgery (Figure 1). The 3 amino acids that changed the most were glutamate, leucine and isoleucine. Multiple comparison analysis showed that most of the changes in circulating amino acid concentrations occurred within the first year. Notably, glutamate levels decreased progressively up to 12 months after surgery and remained stable afterward. Leucine and isoleucine concentrations decreased at 4 months after surgery and remained stable up to 36 months after.

**Figure 2:**
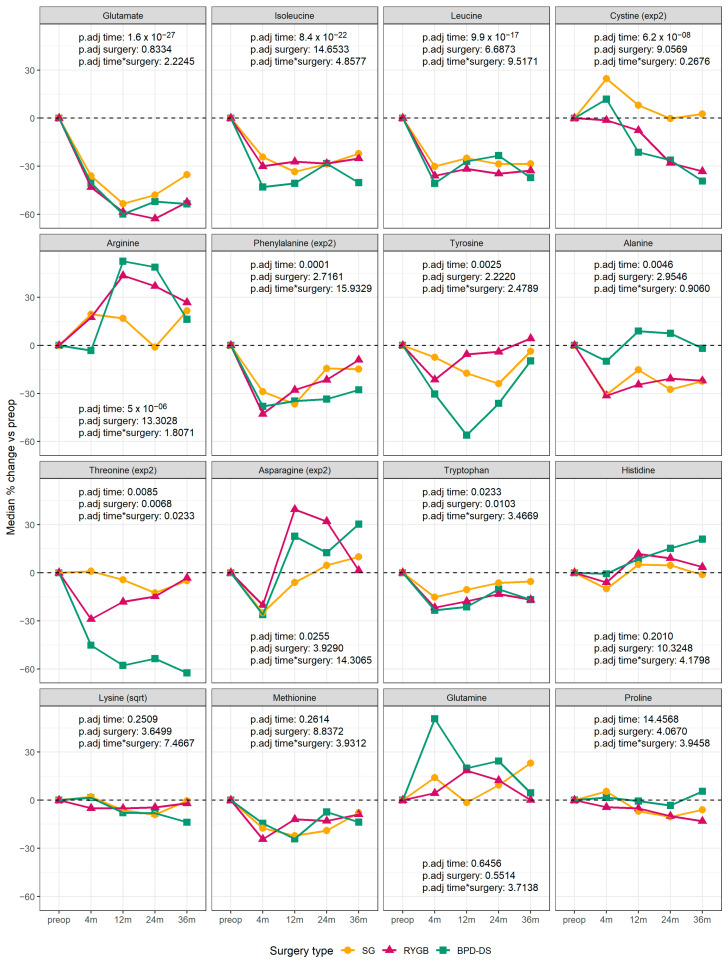
Median changes in circulating amino acids by bariatric surgery type and by time point. The data are expressed as the median percent change versus the baseline. The *p*-values are the result of a mixed linear model fitted using individuals as random effect and time, surgery type and the interaction between time and surgery as fixed effects. The variables were transformed when needed to comply with the test assumptions. The *p*-values were adjusted using Bonferroni’s correction. Yellow circles: sleeve gastrectomy (SG); pink triangles: Roux-en-Y gastric bypass (RYGB); green squares: biliopancreatic diversion with duodenal switch (BPD-DS).

To study the effect of surgery type on the changes in circulating amino acid concentrations after bariatric surgery, we used mixed linear models with time, surgery type and time-by-surgery type interaction as fixed terms. There was a significant interaction between time and surgery type for threonine level, with patients receiving BPD-DS showing a much steeper decrease than the other two groups (Figure 2).

In a mixed linear model with time, type of surgery and BMI as fixed terms, circulating levels of leucine, isoleucine, cystine, alanine, phenylalanine and glutamate were significantly associated with time (all *p*-values under Bonferroni’s adjusted value of 0.05/16). This indicates that changes in the concentrations of these amino acids after bariatric surgery are partially independent of the type of surgery and of the weight loss. Similar results were obtained when using waist circumference instead of BMI in the model.

### 3.3. Association Between Changes in Amino Acid Levels and Changes in Adiposity/Metabolic Variables

**Figure 3:**
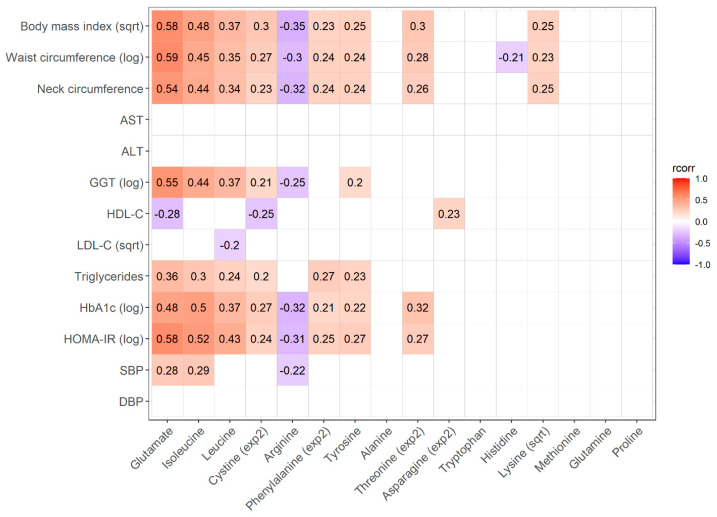
Repeated-measure correlations between circulating amino acids and anthropometric/metabolic variable changes following bariatric surgery. Values are repeated-measure correlations (also called intra-individual correlations, rcorr [18]). Only significant results are shown based on Bonferroni-adjusted *p*-value of 0.05/16 (*p* = 0.003125). Variables were transformed when needed to comply with test assumptions. AST: aspartate aminotransferase; ALT: alanine aminotransferase; GGT: gamma-glutamyl transferase; HDL-C: high-density lipoprotein cholesterol; LDL-C: low-density lipoprotein cholesterol; HbA1c: glycated hemoglobin; HOMA-IR: homeostatic model assessment of insulin resistance; SBP: systolic blood pressure; DBP: diastolic blood pressure.

Figure 3 shows the intra-individual correlations between changes in circulating amino acid levels and adiposity/metabolic variables after bariatric surgery. Changes in adiposity variables (BMI, WC and neck circumference) as well as hemoglobin A1c (HbA1c) and the HOMA-IR index were correlated with changes in the concentrations of at least half of the circulating amino acids examined. Among all of the amino acids, circulating glutamate concentration showed the strongest intra-individual correlations with BMI, WC, gamma-glutamyl transferase (GGT), high-density lipoprotein cholesterol (HDL-C), triglycerides and the HOMA-IR index.

### 3.4. Association Between Circulating Amino Acids and Weight Regain

**Figure 4:**
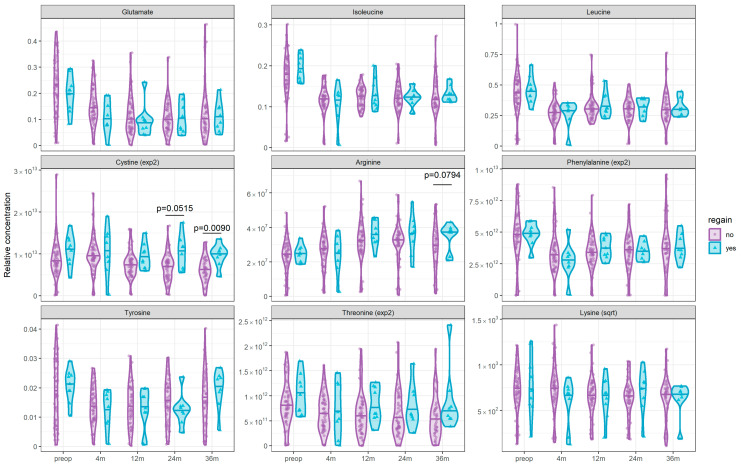
Comparison of circulating amino acids between patients who regained weight after bariatric surgery and those who did not. We defined weight regain as weight gain of more than 10 kg after reaching nadir (lowest weight). Blue triangles and violin plots represent patients who regained weight after nadir (*n* = 9) and purple circles and violin plots represent patients who maintained their weight loss (*n* = 54). Violin plots show distribution of circulating amino acids, middle lines correspond to median and triangles/circles are individual observations. Groups were compared using a two-tailed *t*-test. *p*-values were not adjusted since statistical power was already limited by unequal group sizes, and therefore, they need to be interpreted with caution. *p* values below 0.1 are shown.

To further explore the potential clinical relevance of the intra-individual correlation between anthropometric variables and circulating amino acids, we identified individuals who regained weight after reaching nadir and compared their circulating amino acid profiles with those of patients who maintained their weight loss. Nine participants regained more than 10 kg after reaching nadir, with an average of 14.8 ± 7.6 kg weight regain between nadir and 36 months post-op. There was no significant difference in pre-surgery characteristics between patients who regained weight and those who maintained their weight loss after nadir. We compared the circulating amino acids significantly associated with BMI in intra-correlation analyses between patients who regained weight and those who did not at each time point. Participants who regained weight had significantly higher circulating cystine levels at 36 months post-surgery (Figure 4). There were also trends towards regainers having higher cystine levels at 24 months post-op and higher arginine levels at 36 months post-op.

### 3.5. Association Between Circulating Concentrations and Dietary Intakes of Amino Acids

We used Spearman’s rank correlations to assess the associations between the circulating levels and dietary intakes of amino acids before the surgery as well as 4 months and 12 months after the operation in a subgroup of patients (*n* = 21 at preop and 4 months, *n* = 23 at 12 months). At all time points studied, none of the circulating amino acid levels were associated with their respective dietary intake.

## 4. Discussion

In this study, we aimed to determine the effect of bariatric surgery on circulating amino acids, with an emphasis on changes beyond 1 year and the differences between SG, RYGB and BPD-DS. We observed that the majority of circulating amino acids (11 out of 16 measured) changed significantly after surgery, with the greatest change occurring for glutamate, leucine and isoleucine. Most of the changes in amino acid profiles were achieved as soon as 4 months post-surgery. The changes in circulating threonine levels were different according to the surgical procedures, whereas the changes in other plasma amino acids were not. Changes in many circulating amino acids were correlated with weight loss and improvements in some metabolic parameters.

Our results are consistent with studies of metabolomic changes after bariatric surgery, which often report that branched-chained amino acids (BCAAs, namely leucine, isoleucine and valine) as well as aromatic amino acids (phenylalanine, tryptophan and tyrosine) decrease after these interventions [11]. The literature on the effect of bariatric surgery on circulating glutamate is scarcer and more discordant. For example, Aasheim et al. reported a significant decrease in circulating glutamate levels 12 months after gastric bypass or duodenal switch in 60 participants [20]. On the other hand, Nicolleti et al. reported a decrease in circulating glutamate levels 6 months after RYGB followed by normalization at 12 months in 30 patients [21]. In the present study, circulating glutamate was the amino acid with the most significant decrease after surgery (Figure 1) and this decrease was the most strongly correlated with improvements in adiposity and metabolic markers such as triglycerides, HDL-C, the HOMA-IR index and HbA1c (Figure 3). Considering these results, as well as the literature showing that glutamate is strongly associated with central adiposity [15], we propose that additional studies should be performed to determine the implication of circulating glutamate in metabolic health.

The mechanisms by which amino acids decrease after bariatric surgery have not been completely elucidated. The fact that most circulating amino acids were significantly affected by bariatric surgery suggests that the mechanism might involve global changes simultaneously affecting the metabolism of many amino acids. As an example, the liver–alpha-cell axis describes the ability of glucagon to increase hepatic amino acid catabolism and the feedback action of circulating amino acids to increase glucagon synthesis and secretion by the pancreas [22]. Studies have demonstrated that the liver–alpha-cell axis is altered in the context of liver steatosis; the liver is resistant to glucagon-stimulated amino acid catabolism, resulting in increased amino acid levels [23]. All the participants in our study had liver steatosis, at varying degrees, before bariatric surgery (Table 1). Although we did not assess liver histology after the surgery, other investigators have reported that bariatric procedures decrease liver steatosis significantly [24]. Therefore, the changes in circulating amino acid concentrations that we observed could be the result of an improvement in liver health and normalization of the liver–alpha-cell axis. Other mechanisms specific to some amino acid classes could also be responsible. For example, previous studies have demonstrated that bariatric surgery increases the expression of genes coding for enzymes implicated in BCAA catabolism in adipose tissue [25].

Although repeated-measure correlations showed that changes in most circulating amino acids are strongly correlated with a decrease in adiposity, we found that out of 11 circulating amino acids significantly affected by bariatric surgery, 6 were still significantly associated with time when adjusted for surgery type and BMI. This suggests that mechanisms other than weight loss could contribute to the changes in circulating amino acids observed. This is supported by the study by Lafferère et al., who compared the changes in the metabolomic profiles of patients after 10 kg of weight loss through either RYGB or a dietary intervention [26]. They reported that total amino acid levels decreased only after bariatric surgery. Weight-loss-independent mechanisms for the metabolic improvement associated with bariatric surgery, such as changes in the microbiota, bile acids and gut hormones [10], may be involved in the observed changes in circulating amino acids.

Dietary intakes change significantly after bariatric surgery, due to the restriction of the stomach’s capacity, gastrointestinal symptoms and changes in food preferences [27]. Therefore, it has been postulated that changes in dietary amino acid intakes could explain the changes in circulating amino acids after surgery [11]. In our exploratory analysis of the available dietary data from our cohort, we found no significant association between circulating levels and dietary intakes of amino acids. For glutamate in particular, dietary intakes are unlikely to affect circulating levels, as ~95% of dietary glutamate is oxidized in enterocytes and never reaches the portal vein [28].

Whether the decrease in circulating amino acids is causally implicated in the metabolic improvements following bariatric surgery remains uncertain. Mendelian randomization analyses have demonstrated that plasma levels of BCAAs are associated with a small but significant increase in the risk of developing type 2 diabetes [29]. Similar analyses on other circulating amino acids are lacking.

The potential of circulating amino acids as biomarkers of relevant clinical outcomes is also unclear. In the present study, we explored the association between circulating amino acids and weight regain. We found that only cystine was significantly different between participants who regained weight after nadir compared to those who did not. Moreover, the difference was seen at 36 months post-surgery (with a trend at 24 months). Therefore, these results reflect the correlation between cystine and body weight but would not be useful to predict which patients will regain weight. Because only nine participants regained weight, we had limited power to detect significant differences, and this result should be interpreted with caution. However, it suggests that the change in circulating amino acids after bariatric surgery is interesting to study the physiological changes induced by the intervention, but not necessarily as a biomarker of clinical outcomes. In this study, hydrophilic interaction liquid chromatography was used to measure amino acids in plasma samples. This approach has been shown to be simple, accurate and particularly relevant when amino acids are measured as part of a broader metabolomic panel [30]. Other methods have been shown to accurately measure circulating amino acids, notably derivatization (pre- or post-column), gas chromatography, chiral analysis and capillary electrophoresis [31].

The strengths of this study are the matching of participants between surgery types, the presence of BPD-DS surgery (which is rarely studied [32]) and the follow up of 3 years. The study limitations include the fact that the surgical groups could not be matched for BMI, the small number of participants in the preliminary analysis of dietary data and the lack of measurements of elements of the liver–alpha-cell axis, such as post operative liver histology or plasmatic glucagon. The study was also not a randomized trial, due to requirements in the nutritional follow-up of BPD-DS patients, which are different from those of SG and RYGB patients. Finally, we did not assess the matrix effect and the stability of the amino acids measured, but we used internal standards to mitigate these issues.

## Figures and Tables

**Table 1:** Sampe characteristics at baseline by surgery type.

Variable (Unit)	*n*=	SG	RYBP	BPD-DS	*p*-Value
Age (years)	63	51.7 (44.8–55.3)	51.5 (44.0–54.8)	51.0 (43.9–55.7)	0.9471
Sex (female/male)	63	14/7	14/7	14/7	1.0000
Body mass index (kg/m^2^)	62	44.2 (41.8–48.2)	40.8 (38.4–43.0)	49.3 (45.0–50.6)	3.6 × 10^−6^
Waist circumference (cm)	62	132 (122–141)	128 (125–137)	141 (135–151)	0.0126
Neck circumference (cm)	62	44.0 (41.0–46.0)	42.0 (39.0–47.0)	44.0 (42.5–48.0)	0.4337
ALT (U/L)	63	27.0 (17.0–36.0)	32.0 (23.0–44.0)	29.0 (22.0–36.0)	0.3783
GGT (U/L)	63	27.0 (21.0–45.0)	36.0 (22.0–56.0)	36.0 (24.0–42.0)	0.6208
HDL-C (mmol/L)	63	1.11 (0.86–1.22)	1.07 (0.88–1.20)	1.05 (0.95–1.39)	0.7518
LDL-C (mmol/L)	61	1.98 (1.68–2.38)	1.98 (1.51–2.38)	1.77 (1.51–2.19)	0.3488
Triglycerides (mmol/L)	63	1.86 (1.20–2.37)	1.52 (1.33–2.07)	1.59 (1.16–2.61)	0.8992
HbA1c (%)	63	6.40 (5.90–7.00)	7.10 (6.70–7.30)	6.90 (6.40–7.50)	0.0537
HOMA-IR	62	13.94 (11.44–17.61)	11.97 (7.67–16.75)	14.00 (7.88–26.79)	0.7092
SBP (mmHg)	63	138 (137–147)	144 (131–148)	145 (138–154)	0.1700
DBP (mmHg)	63	78.0 (71.0–90.0)	82.0 (74.0–95.0)	83.0 (77.0–86.0)	0.5935
Steatosis grade (0/1/2/3)	57	0/13/5/2	0/9/5/4	0/15/2/2	0.4079
NASH (no/yes)	51	5/13	5/10	6/12	0.9199
Fibrosis grade (0/1/2/3/4)	57	5/7/4/4/0	5/9/3/1/0	3/10/3/3/0	0.8084

Results presented as median (Q1–Q3) unless stated otherwise. *p*-value from Kruskal–Wallis test. SG: sleeve gastrectomy; RYGB: Roux-en-Y gastric bypass; BPD-DS: biliopancreatic diversion with duodenal switch; ALT: alanine aminotransferase; GGT: gamma-glutamyl transferase; HDL-C: high-density lipoprotein cholesterol; LDL-C: low-density lipoprotein cholesterol; HbA1c: glycated hemoglobin; HOMA-IR: homeostatic model assessment of insulin resistance; SBP: systolic blood pressure; DBP: diastolic blood pressure; NASH: non-alcoholic steatohepatitis. Reference values for clinical variables presented can be found in Supplementary Table S3.

## Data Availability

The datasets presented in this article are not readily available because the data are part of an ongoing study. Requests to access the datasets should be directed to the corresponding author.

## Supplementary Materials

The following supporting information can be downloaded at: https://www.mdpi.com/article/10.3390/metabo15050297/s1, Figure S1: Example of extracted ion chromatogram for the amino acids reported in Table S3. Top chromatogram is the amino acids standard and bottom chromatogram is from a QC pool plasma sample; Table S1: Mobile phase gradient; Table S2: Mass and retention time measured for 16 amino acids measured [33]; Table S3: Reference values for clinical variables presented in Table 1 [34,35,36,37].
