# Supplementary material for: Circulating Amino Acid Changes Three Years After Bariatric Surgery
Source: Metabolites. 2025 Apr 30;15(5):297. doi: 10.3390/metabo15050297 (PMC12112989; doi:10.3390/metabo15050297)
Supplement: Supplementary file 1 [file metabolites-15-00297-s001.zip › metabolites-3242632-supplemental Figure S1.pdf]

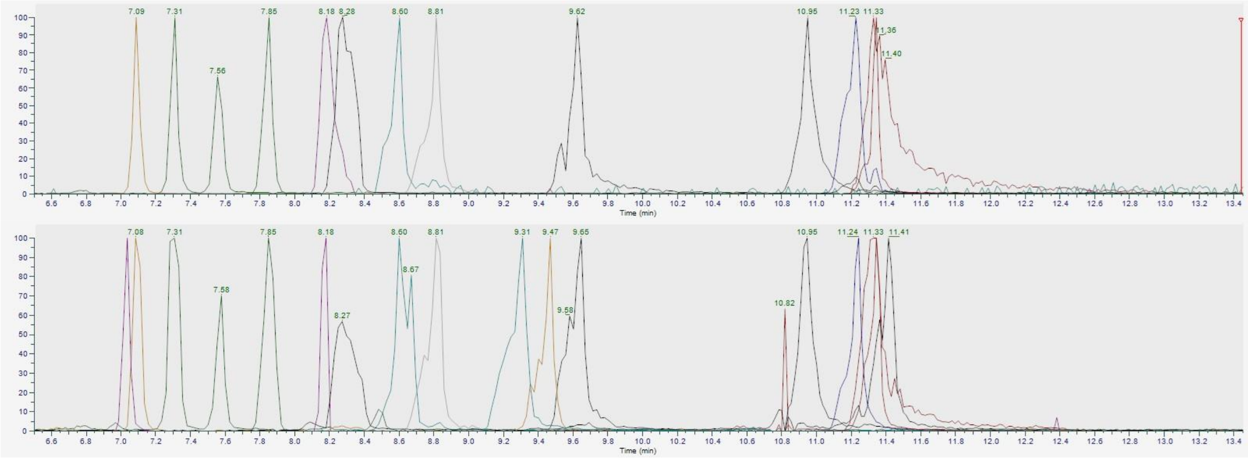

Supplemental Figure S1. Example of extracted ion chromatogram for the amino acids reported in Supplemental Table 3. Top chromatogram is the amino acids standard and bottom chromatogram is from a QC pool plasma sample.
